# Cancer drug resistance induced by EMT: novel therapeutic strategies

**DOI:** 10.1007/s00204-021-03063-7

**Published:** 2021-05-18

**Authors:** Javier De Las Rivas, Anamaria Brozovic, Sivan Izraely, Alba Casas-Pais, Isaac P. Witz, Angélica Figueroa

**Affiliations:** 1 Bioinformatics and Functional Genomics Group, Cancer Research Center (CiC-IBMCC, CSIC/USAL/IBSAL), Consejo Superior de Investigaciones Científicas (CSIC), University of Salamanca (USAL), Salamanca, Spain; 2 Division of Molecular Biology, Ruđer Bošković Institute, Bijenička 54, 10000 Zagreb, Croatia; 3 Shmunis School of Biomedicine and Cancer Research, George S. Wise Faculty of Life Sciences, Tel-Aviv University, Tel Aviv, Israel; 4 Epithelial Plasticity and Metastasis Group, Instituto de Investigación Biomédica de A Coruña (INIBIC), Complexo Hospitalario Universitario de A Coruña (CHUAC), Sergas, Spain; 5 Universidade da Coruña (UDC), Coruña, Spain

**Keywords:** Epithelial plasticity, Cancer, Therapy resistance, Tumour microenvironment

## Abstract

Over the last decade, important clinical benefits have been achieved in cancer patients by using drug-targeting strategies. Nevertheless, drug resistance is still a major problem in most cancer therapies. Epithelial-mesenchymal plasticity (EMP) and tumour microenvironment have been described as limiting factors for effective treatment in many cancer types. Moreover, epithelial-to-mesenchymal transition (EMT) has also been associated with therapy resistance in many different preclinical models, although limited evidence has been obtained from clinical studies and clinical samples. In this review, we particularly deepen into the mechanisms of which intermediate epithelial/mesenchymal (E/M) states and its interconnection to microenvironment influence therapy resistance. We also describe how the use of bioinformatics and pharmacogenomics will help to figure out the biological impact of the EMT on drug resistance and to develop novel pharmacological approaches in the future.

## Background

At present, one of the most important challenges in oncology is to overcome therapy resistance, as it is a persistent problem for cancer patient management. Frequently, patients with resistance also develop more metastases, and given that metastasis is the major cause of cancer-related deaths in human carcinomas, it is important to overcome therapy resistance by using new targeted-therapy strategies. Therapy resistance not only includes the traditionally well-established innate and acquired tumour drug resistance, but it also includes resistance to treatment such as chemo or radiotherapy, immune- and targeted-therapies (Burrell and Swanton 2016; Assaraf et al. 2019; Vasan et al. 2019). Important molecular mechanisms involved in drug resistance have been well determined by the effect of a decreased drug uptake by altered influx transporters, an increased drug efflux by the overexpression of multidrug-resistance (MDR) efflux transporters or an altered expression of anti-apoptotic proteins (Assaraf et al. 2019). However, still limited understanding of the molecular mechanisms involved in therapy resistance has been elucidated. Epithelial-to-mesenchymal transition (EMT) has emerged as a major contributor to therapy resistance. EMT is a highly conserved cellular program that allows polarized, immobile epithelial cells to transform into mesenchymal, mobile cells because of the loss of apico-basal polarity, the loss of cell–cell contacts, the reorganization of the actin cytoskeleton, and the ability to invade the extracellular matrix as an individual cell. EMT is related to tumour progression, metastasis and mediates resistance to conventional therapies and small-molecule targeted inhibitors (Thiery et al. 2009; Chaffer et al. 2016; Yang et al. 2020). Important studies using tumour cell lines demonstrate the implication of EMT in resistance driven by radio- or chemotherapy (Inoue et al. 2002; Olmeda et al. 2007). However, insufficient in vivo information is available mainly due to the absence of suitable in vivo models and limited human samples analyzed to perform comprehensive studies. On the other hand, it is important to highlight that intermediate epithelial and mesenchymal (E/M) phenotypic states coexists in a carcinoma, therefore different subpopulation are found, increasing the level of plasticity within the tumour (Yang et al. 2020). Although the influence of these intermediate E/M states on resistance to anticancer therapeutics drugs is not fully understood, pharmacogenomics approaches impact on this relevant aspect. Moreover, an important link between EMT and tumour microenvironment has arisen as a state of the art of research in oncology, highlighting the need of personalized treatments for individual cancer patients (Shibue and Weinberg 2017; Maman and Witz 2018; Gupta et al. 2019; Recasens and Munoz 2019; Boumahdi and de Sauvage 2020). Given the recent outstanding contributions published on the importance of tumour microenvironment and EMT in multidrug resistance (MDR), this issue will be not further discussed (Erin et al. 2020). In this review, we will go in depth into the molecular mechanism by which EMT induce therapy resistance and how the microenvironment contribute to this process. Moreover, future perspectives on bioinformatic and pharmacological approaches to overcome therapies resistance will be also discussed.

## Epithelial-to-mesenchymal transition and tumour resistance: evidences in vitro, in vivo and in clinical studies

The cancer EMT program is a cellular and molecular process by which epithelial tumour cells lose cell–cell contacts and apico–basal polarity, acquiring mesenchymal characteristics (Brabletz et al. 2018; Yang et al. 2020). Importantly, EMT is a highly dynamic and reversible process, on which mesenchymal cells can revert to epithelial phenotype by mesenchymal-to-epithelial transition process (MET) (Thiery et al. 2009). Intermediate cellular states, E/M hybrid phenotypes, coexist within the tumour harboring high degree of epithelial–mesenchymal plasticity (EMP). The epithelial-to-mesenchymal plasticity is tightly regulated at a transcriptional, post-transcriptional and post-translational level, with important clinical implications (Sabbah et al. 2008; Aparicio et al. 2013, 2015). The loss of expression of E-cadherin protein at cell–cell contacts is a hallmark of the EMT, which is accompanied by the downregulation of other epithelial proteins, such as cytokeratins, claudins, and the upregulation of mesenchymal markers, such as *N*-cadherin, Vimentin or Fibronectin (Nieto et al. 2016; Brabletz et al. 2018; Yang et al. 2020). Moreover, several transcription factors (TFs) are involved in the EMT, as the repressors of E-cadherin promoter including the Snail/Slug family, Twist, Zeb1 and Zeb2 (Batlle et al. 2000; Cano et al. 2000). The loss of E-cadherin is also regulated by posttranscriptional regulators (such as miR-200 family or RNA-binding proteins) or by posttranslational regulators (such as CK1 or Hakai) (Park et al. 2008; Gregory et al. 2008; Sarkar et al. 2010; Wang et al. 2010; Aparicio et al. 2013).

Although many publications have reported the implication of EMT on cancer metastasis, important articles support that EMT program is dispensable in this process (Arumugam et al. 2009; Fischer et al. 2015; Zheng et al. 2015). However, the relationship between EMT and therapy resistance is increasingly established. Indeed, the link between EMT and cancer stemness and their influence on drug resistance has been recently reported, therefore this topic will be not recapitulated in detail (Koren and Fuchs 2016; Chaffer et al. 2016; Shibue and Weinberg 2017; Dongre and Weinberg 2019). The general mechanism regarding EMT-associated drug resistance is related to increased drug efflux, slow cell proliferation and avoiding apoptosis signaling pathways. Moreover, avoiding immune response is another important mechanism by which EMT contributes to therapeutic resistance, by altering expression of molecules involved in immunosuppression or immunoevasion (Shibue and Weinberg 2017; Gupta et al. 2019; Dongre and Weinberg 2019). Although EMT can have an impact on drug resistance in several preclinical models (Shibue and Weinberg 2017; Gupta et al. 2019), the understanding of the molecular mechanism is poorly understood as recapitulated below. Many publications highlight the impact of EMT in vitro, in vivo and in clinical specimens. It is reported the implication of transcriptional or posttranslational EMT-related regulators in resistance to anticancer therapeutic drugs (Kajita et al. 2004; Yauch et al. 2005; Olmeda et al. 2007; Saxena et al. 2011; Shibue and Weinberg 2017; Weng et al. 2019; Dongre and Weinberg 2019; Boumahdi and de Sauvage 2020). Here, we will discuss recent EMT studies in different cancer models.

Perhaps the most controversial studies in this field were reported in vivo, using transgenic mice models (Fischer et al. 2015; Zheng et al. 2015). Using a EMT lineage tracing a triple transgenic mice of breast cancer, it was demonstrated that inhibition of EMT by overexpression of miR-200a and, in consequence, the abolition of the EMT-TFs Zeb1 and Zeb2, abrogated chemoresistance to cyclophosphamide (Fischer et al. 2015). Additionally, by deleting Snail or Twist TFs in genetically engineered mouse models of pancreatic ductal adenocarcinoma resulted in an enhanced expression of nucleoside transporters in tumours, which in turn increased the sensitivity to gemcitabine treatment (Zheng et al. 2015). Although both studies were very controversial as the contribution of EMT on cancer metastasis was not supported, this affirmation was later argued (Aiello et al. 2017; Ye et al. 2017). However, the involvement of the EMT in drug resistance was very well demonstrated in these two in vivo preclinical models, and the implication of EMT in chemoresistance underscored the potential benefit of combining EMT inhibition with chemotherapy for the cancer treatment.

**Fig. 1:**
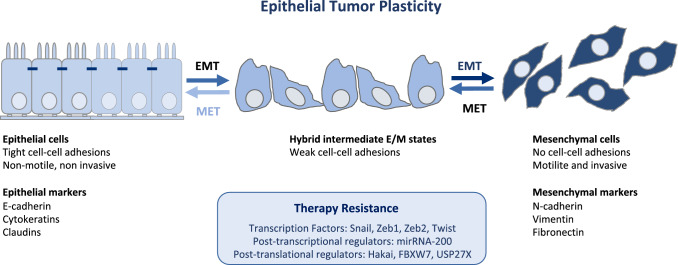
Targeting cancer epithelial tumour plasticity to overcome resistance. Tumour cells with epithelial phenotype can undergo epithelial-to-mesenchymal transition program at primary tumour site. Epithelial cells loose cell–cell contacts and aquiere invasive and migratory capabilities. The existence of intermediate epithelial/mesenchymal marker proteins in cancer cells with partial E/M hybrid phenotype is associated with increased cellular plasticity and stemness. Several transcription factors, post-trasncriptional and post-translational regulators of the EMT are implicated in therapy resistance

Apart from these two important contributions using transgenic mice models, the majority of data linking EMT to chemoresistance is supported by in vitro studies, xenograft tumours using athymic mice, and in clinical specimens. Perhaps, lung cancer is one the best type of cancer on which the link between EMT and resistance to therapy is well documented. It has been demonstrated that targeting FGFR prevents the development of EMT-mediated resistance in EGFR mutant NSCLC (Raoof et al. 2019). On the other hand, epigenetic silencing of miR-483-3p has been reported to promote acquired gefitinib resistance and EMT in EGFR-mutant NSCLC (Yue et al. 2018). Many publications underscore the implication of EMT-TFs in drug resistance (Fig. 1). For instance, the overexpression of Snail and Slug has been reported to induce gefitinib resistance in EGFR-mutant lung cancer cell lines (Lee et al. 2017a). Moreover, PAX6 has been demonstrated to induce EMT and cisplatin resistance through the regulation of Zeb2 expression (Wu et al. 2019a). The upregulation of the EMT-associated gene AXL, has been described to predict acquired resistance to EGFR-TKI osimertinib (Namba et al. 2019). The molecular mechanism proposed for this therapy resistance is the participation of cell stem characteristics, the repression of the proapoptotic protein Bcl-2-like protein 11 or the chromatin remodeling driven by EMT-TFs (Sayan et al. 2009; Song et al. 2018). Moreover, MET-driven EMT has also been demonstrated to induce chemoresistance. On the one hand, cisplatin-resistant NSCLC cell lines showed MET overexpression compared to parental ones, which is accompanied by and increased expression of *N*-cadherin, Vimentin, Zeb1 and Snail, and a decreased expression of E-cadherin. On the other hand, mir-206-mediated MET downregulation not only reversed EMT but also sensitized resistant NSCLC cells to cisplatin (Chen et al. 2016).

In breast cancer, it was described that the overexpression TFs such as Twist, Snail, and FOXC2 increases the promoter activity of ABC transporters, indicating that EMT inducers are novel regulators of ABC transporters. Therefore EMT-TFs are proposed as novel strategies to treat metastasis and the associated drug resistance (Saxena et al. 2011). Importantly, it has been elucidated that intermediate E/M phenotypes in breast cancer cells are more effective in developing drug resistance and metastasis than when a complete mesenchymal state has occurred (Jolly et al. 2019). One of the molecular mechanisms proposed for this resistance in mesenchymal-like triple-negative breast cancer cells is due to the expression of ITGB4 + in intermediate states, regulated by Zeb1 through its repression on Tap63α expression, a protein that promotes ITGB4 expression (Bierie et al. 2017). Another important example of the implication of EMT-TFs in drug resistance was reported in normal and transformed human mammary epithelial on which induction of Twist overexpression or E-cadherin inhibition confer resistance to paclitaxel and doxorubicin. On the other hand, Snail confers resistance to docetaxel and gemcitabine in basal-like breast cancer MDA-MB-231 cells. However, breast cancer cells with mesenchymal characteristics are sensitive to paclitaxel. Indeed, it has been demonstrated that induction of EMT activates PERK-eIF2α and sensitizes cells to agents that perturb endoplasmic reticulum function, which shows a new vulnerability of cancer cells that undergo EMT, consisting in the sensitivity to endoplasmic reticulum stress (Olmeda et al. 2007; Gupta et al. 2009; Feng et al. 2014). Importantly, by using in vitro and in xenograft models, the link between EMT and endocrine therapy resistance in luminal breast cancer has been reported. Indeed, when estrogen receptor alpha gene (ESR1) fusion proteins is expressed in breast cancer cell lines it promotes an estrogen-independent activation of EMT by Snail upregulation and E-cadherin downregulation (Lei et al. 2018). Other examples of the link between EMT and therapy resistance have been shown in prostate and ovarian cancer. Indeed, prostate tumour resistance to cabazitaxel can be overcome by antiandrogen-mediated EMT-MET in androgen-sensitive tumours but not in metastatic castration-resistant prostate cancer patients, who frequently develop therapeutic resistance to taxane chemotherapy and antiandrogens. On the other hand, Lysyl oxidase-like 2 (LOXL2), a protein that induces EMT, is involved in radiotherapy resistance in prostate cancer cells and in xenografts mice model (Cano et al. 2012; Martin et al. 2016). In ovarian cancer, the EMT-TFs Snail and Slug drive chemo and radioresistance through the p53-driven apoptosis and regulation of stem properties (Kurrey et al. 2009). In colorectal cancer, miR-128-3p reverses oxaliplatin resistance in colorectal cancer through the downregulation of Bmi1 and MRP5, two genes involved in oxaliplatin-induced EMT (Liu et al. 2019).

The involvement of the post-translational EMT regulators in drug resistance has been described (Fig. 1). It was demonstrated that early stages of EMT involve a post-translational downregulation of E-cadherin, whereas loss of E-cadherin via transcriptional repression is a late event in EMT (Janda et al. 2006). As previously mentioned, the E3 ubiquitin-ligase Hakai is a posttranslational regulator of E-cadherin stability (Fujita et al. 2002; Aparicio et al. 2012). Hakai is upregulated in gefitinib-resistant NSCLC cells that acquired EMT characteristics. Moreover, an increase of Hakai and a decrease in E-cadherin expression is also detected in gefitinib-resistant clinical cancer samples and lung cell lines. This event was reversed by the dual action of histone deacetylase (HDAC) and 3-hydroxy-3-methylglutaryl coenzyme A reductase (HMGR) inhibitor, JMF3086. Indeed, JMF3086 inhibited the Src/Hakai and Hakai/E-cadherin interaction reverting E-cadherin expression and reducing Vimentin and stemness to restore gefitinib sensitivity (Weng et al. 2019; Boumahdi and de Sauvage 2020). This study not only underscores the implication of the posttranslational regulators of the EMT in gefitinib-resistance (beyond EGFR mutations per se), but also draws the attention for therapeutic targeting of Hakai to block EMT and overcome chemoresistance in combination with chemotherapy. In fact, a recent study identified Hakin-1 as a novel specific small-molecule inhibitor against Hakai, emerging as an effective therapeutic agent for EMT inhibition (Martinez-Iglesias et al. 2020). Given the mechanism of action of Hakai, it is expected that different types of carcinomas, such as colorectal cancer or lung cancer, may benefit with this therapy (Figueroa et al. 2009; Aparicio et al. 2015; Castosa et al. 2018). Another important posttranslational mechanism that may impact therapy resistance, is described in colorectal cancer (Díaz and de Herreros 2016; Li et al. 2019). The transcription factor Zeb2 is a substrate for the F-Box E3 ubiquitin-ligase FBXW7 in intestinal stem cells upon GSK3β phosphorylation. In mouse and human colorectal cancer cell lines, the axis Zeb2/FBXW7 induces EMT and metastasis, and it is linked to chemoresistance (Díaz and de Herreros 2016; Li et al. 2019). Other important proteins involved in ubiquitin–proteasome pathway was recently reported to be involved in breast and pancreatic cancer cells (Lambies et al. 2019). It was shown that TGF-β-induced EMT activates the deubiquitinase USP27X, which stabilize Snail protein. In the absence of USP27X, Snail is degraded and the sensitivity to cisplatin is increased, opening new therapeutic strategies to overcome chemoresistance (Lambies et al. 2019).

## Regulation of the epithelial-to-mesenchymal transition by tumour microenvironment

Solid tumours are a cellular ecosystem termed tumour microenvironment (TME). In addition to tumour cells the cellular content of the TME is composed of resident and infiltrating non-tumour cells including endothelial cells, fibroblasts, various types of lymphatic cells such as T, B and NK cells; myeloid cells such as macrophages and granulocytes and others. A major component of the acellular fraction of the TME is the extra cellular matrix (ECM), a network of multiple categories of macromolecules. Other TME constituents are soluble products of the microenvironmental cells such enzymes, cytokines, chemokines and antibodies. The metabolome of the TME very often differs from the corresponding normal organ and hypoxia characterizes the TME of most solid tumours. Drugs may also be present in the TME of treated tumour bearers (Maman and Witz 2018). The TME is an arena for dynamic and constant interactions between tumour cells, their molecular products and host-derived cells and molecules. The reciprocal tumour-host interactions lead to an evolving phenotype reprograming of both interaction partners and may culminate in metastasis and therapy resistance (Dalton 1999; Morin 2003; Correia and Bissell 2012; Maman and Witz 2018).

The survival, propagation and the progression of cancer cells towards metastasis depend on intrinsic properties of the cancer cells as well as on cross-talk with their microenvironment (Klein-Goldberg et al. 2014; Maman and Witz 2018). The spread of cancer cells from the primary site to secondary organ sites and then the establishment of new cancer lesions in these sites is a sequential multistep process. Each step of the metastatic cascade is jointly controlled by tumour-intrinsic factors as well as by those originating in the TME (Klein-Goldberg et al. 2014; Maman and Witz 2018). One of the initial phases of the metastatic cascade is driven by the activation of the EMT program that confers to tumour cells the capacity to invade neighboring tissues. Then, cells reach the circulation, spread throughout the body and subsequently metastasize to specific organs. EMT program is triggered in response to TME-derived paracrine signals emitted from resident or infiltrating non-tumour cells such as fibroblasts, macrophages or immunocytes (Lamouille et al. 2014; Brabletz et al. 2018; Yang et al. 2020). The molecular program that drives EMT functions via miocroenvironmental multi-signaling pathways that cooperate and cross talk to each other. Among these are members of the TGF superfamily, VEGF, HGF, HIFs, Notch and Wnt, ECM components and microRNAs to mention but a few (Thiery et al. 2009; Lindsey and Langhans 2014; Mudduluru et al. 2015; Ye and Weinberg 2015; Zhang et al. 2016; Dongre and Weinberg 2019). These, sometimes converging, signaling pathways upregulate several EMT-transcription factors such as Twist, Zeb1 and Snail (Cano et al. 2000; Peinado et al. 2007; Taube et al. 2010).

**Fig. 2:**
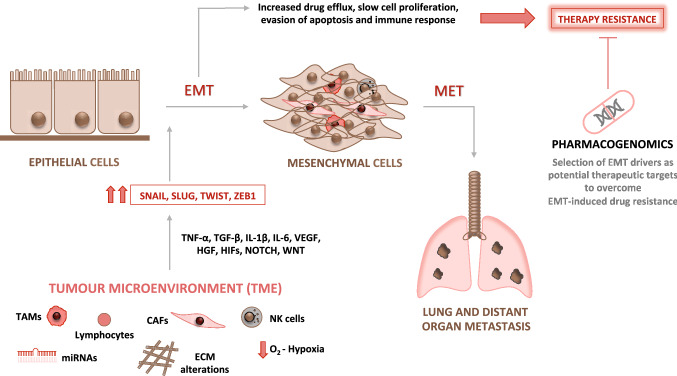
Microenvironment drivers of the EMT as potential therapeutic target to overcome therapy resistance. Several microenvironment factors such as tumour associated macrophages (TAMs), cancer associated fibroblasts (CAFs), alterations in the extracellular matrix (ECM), hypoxic conditions, inflammatory and immune cells are EMT-drivers. These cells activate several signaling pathways such as TNF-α, TGF-β, IL-1β, IL-6, VEGF, HGF, HIFs, NOTCH and WNT, inducing EMT-transcription factors

These EMT-regulated factors act in concert to alter cellular morphology, promote motility, reprogram ECM, and downregulate tight junctions (Peinado et al. 2007; Wheelock et al. 2008). Importantly, during EMT process distinct intermediate stages are found, existing tumour subpopulations expressing phenotypes ranging from a complete epithelial to a complete mesenchymal one may co-inhabit single solid tumours. The microenvironmental host cells associated with different tumour subpopulations may also vary (Pastushenko et al. 2018). Moreover, it should also be remembered that the mesenchymal-tumour cells generated by the EMT process exert various effects on microenvironmental cells that could impact tumour progression and drug resistance (Nassar and Blanpain 2016; Dongre and Weinberg 2019). The main microenvironmental drivers of EMT will be discussed below (Fig. 2).

### Hypoxia

Hypoxia, characterizing the microenvironment of essentially all solid tumours, is a major driver of EMT. The hypoxia-mediated effects are exerted by Hypoxia-Inducible Factors (HIFs), a family of transcriptional regulators that control functions involved in tumour progression such as extracellular matrix (ECM) remodeling, cell survival and proliferation, metabolism, inflammation and angiogenesis. HIFs also play pivotal functions in the EMT process and drug resistance (Rohwer and Cramer 2011; Balamurugan 2016; Schito and Rey 2017; Joseph et al. 2018). HIF-1, a member of the HIF family, upregulates the expression and activity of several EMT-inducing transcription factors including Twist, Zeb1 and Snail. Each of these factors alone has the capacity to induce EMT (Yang et al. 2008). These factors repress the expression by tumour cells of epithelial-specific proteins such as E-cadherin while inducing the acquisition of a mesenchymal phenotype in these cells. HIF-1 may exert its influence by functioning in concert with other factors. For example, HIF-1 engages in a crosstalk with members of the TGF-β family, being themselves strong inducers of EMT (the role of TGF-β family members in EMT is discussed separately). These 2 interaction partners cooperatively support EMT (Copple 2010).

### The extracellular matrix

The extracellular matrix (ECM), a three-dimensional network that surround the cells in a certain microenvironment, is an important constituent of the TME that provides structural and biochemical support to such cells. Its main functions are to support cell adhesion and inter cellular communication (Hynes and Naba 2012). The ECM is composed of macromolecules, such as integrins, collagen, glycoproteins, glycosaminoglycans and enzymes to name but a few. A review by Tzanakakis provides a detailed account of the ECM constituents that interact with EMT components thereby regulating this process (Tzanakakis et al. 2018). Deregulated ECM remodeling, induced by matricellular proteins, reactive oxygen species, by hypoxia or by proteases, has a meaningful impact on tumour progression especially by being both affected by this process as well as influencing it. ECM remodeling involves alterations in the expression of proteoglycans, a reorganization of the collagen interactome, proteolysis of macromolecules and activation of integrins. These alterations in ECM structure and function drive EMT (Catalano et al. 2013; Nieberler et al. 2017; Paolillo and Schinelli 2019; Brassart-Pasco et al. 2020; Gerarduzzi et al. 2020).

### The inflammatory and immune microenvironment

Tumour promoting and tumour suppressive immunocytes and inflammatory cells as well as their molecular products are abundantly present in the TME (Maman and Witz 2018). The inflammatory cells as well as their secretome have the capacity to induce, drive and maintain EMT (Yan et al. 2018; Fedele and Melisi 2020). Tumour-associated macrophages (TAMs) are the largest non-tumour cell population in the TME. These cells promote tumour progression by secreting the angiogenic cytokine VEGF, and by activating inflammatory pathways via pro-inflammatory cytokines (Noy and Pollard 2014; Ségaliny et al. 2015; Mantovani et al. 2017). TAMs also play a crucial role in the induction and maintenance of EMT (Song et al. 2017), for example by secreting pro-inflammatory cytokines such as TNF-α, TGF-β, IL-1β, IL-6, CCL5 and CCL18. TAMs are involved in the activation of the EMT process by using various modes of action (Suarez-Carmona et al. 2017; Dominguez et al. 2017). Other myeloid cells such as granulocytes or myeloid-derived suppressor cells also induce EMT (Toh et al. 2011; Mayer et al. 2016; Sangaletti et al. 2016). Tumour infiltrating lymphocytes such as regulatory T cells (Kudo-Saito et al. 2009) are also involved in the activation of EMT mainly via secretion of pro-inflammatory and other tumour-promoting cytokines. The cross talk between tumour and NK cells taking place in the microenvironment induces a skewed phenotype in NK cells becoming drivers rather than inhibitors of metastasis. This metastasis-promoting function is implemented via the activation of EMT. The tumour infiltrating NK cells secrete pro-inflammatory cytokines such as IL-6 and activate various matrix metalloproteinases that facilitate tumour invasion (Cantoni et al., 2016; Lee et al. 2017b). EMT may induce immune suppressive properties in cancer cells (Ricciardi et al. 2015; Terry et al. 2017) or modify their immunogenicity resulting either in escape from anti-tumour immune responses or in the generation of new tumour-associated epitopes (Chockley and Keshamouni 2016; Poggi and Giuliani 2016).

### Cancer-associated fibroblasts

Cancer-Associated Fibroblasts (CAFs) together with myeloid-derived cells (mostly macrophages) and to lesser degree endothelial cells are the most abundant non-tumour cells in the TME. CAFs are generated as a response to activation signals delivered to fibroblasts from tumour and non-tumour cells in the TME. Such signals which mediate their function by contact between tumour cells and fibroblast or by soluble factors such as IL-1 or IL-6, induce the CAF phenotype characterized by the expression of α-smooth muscle actin (α-SMA) (Sahai et al. 2020). CAFs promote cancer progression by EMT facilitating functions such as reprograming of ECM and of the phenotype of tumour and of other TME-residing cells. These functions are mediated by cellular contacts between CAFs and tumour cells or other stromal cells or by soluble factors (Choe et al. 2013; Yu et al. 2014; Chen and Song 2019). Among the EMT-enhancing factors released from CAFs are TGF-β and proinflammatory cytokines (Yu et al. 2014; Fang et al. 2018).

### MicroRNAs

MicroRNAs (miRNAs) are small non-coding RNAs that extensively regulate gene expression by binding mRNA thereby inhibiting its translation. This capacity enables miRNAs to function as potent regulators of normal cellular physiology and when aberrantly expressed, also of pathological processes such as cancer progression (Fabian et al. 2010; Markopoulos et al. 2017). EMT is regulated by miRNAs. These molecules downregulate EMT-associated transcription factors, or alternatively act as their functional mediators in the regulation of the EMT process (Abba et al. 2016). As noted above TGF-β, Notch, and Wnt signaling pathways are intimately linked to the EMT process. The discovery of a signature of 30 miRNAs, each regulating all of these 3 pathways and of the target genes of these miRNAs, demonstrated the occurrence of an EMT-promoting cross talk between these pathways (Zoni et al. 2015). Multiple miRNAs including miR-200, miR-34, miR-338-3p and others are involved in the regulation of EMT (Park et al. 2008; Li et al. 2016; Nie et al. 2019). Members of the miR-200 family may serve as prototypes for miRNAs that influence EMT. These miRNAs have been extensively studied for their role as master regulators (suppressors) of EMT.

### TGF-β

TGF-β is a multifunctional cytokine produced by tumour as well as by host-derived cells within the TME (Izraely et al. 2017; Ahmadi et al. 2019). TGF-β regulates various functions of tumour cells and of host-derived cells within the TME by employing TGF-β type I and type II receptors (Heldin and Moustakas 2016). TGF-β can be regarded as a prototype of molecules that exert yin-yang functions with respect to tumourigenesis and tumour progression (Witz 2008). In early phases of tumour progression TGF-β usually functions as a tumour suppressor whereas in later phases it promotes malignancy (Yang et al. 2010; Suriyamurthy et al. 2019) mainly by acting as the main inducer and driver of EMT, leading to tumour progression towards metastasis (Hao et al. 2019). Complexes of TGF-β and its kinase receptors activate the intracellular transcriptional effectors Smad. These, in turn, regulate the expression of EMT-mediating genes Twist, Zeb1 and Snail. Following are some selected studies documenting the role of Smad in TGF-β-mediated EMT (Vincent et al. 2009; Kim et al. 2016; Yu et al. 2016; Yeh et al. 2018; Tong et al. 2020). TGF-β can also induce EMT by altering the mechanical architecture (cytoskeletal remodeling) of cancer cells to a motile phenotype (Gladilin et al. 2019). This Smad-independent process involves the activation of ERK (Lee et al. 2007). Similarly to the TGF-β/HIF-1 cooperation (Copple 2010) TGF-β collaborates with other EMT inducers such as Wnt or Notch to co-stimulate EMT thereby promoting an invasive and pro-metastatic phenotype of tumour cells (Murillo-Garzón et al. 2018).

## Microenvironmental regulation of EMT: influence on drug resistance

Tumour microenvironment, as an important regulator of the EMT, has an impact on therapy resistance. Many publications have highlighted that signals such as growth factors or cytokines originated from tumour stroma may regulate EMT-related drug resistance (Shibue and Weinberg 2017). Between them, one of the extracellular matrix factors secreted by CAFs are TGF-β1 and hyaluronan. The first evidence showing the properties of hyaluronan in cancer resistance was reported in a model of naked rat mole fibroblasts secreting high molecular mass hyaluronan, that hyper sensitize cells to contact inhibition and cell cycle arrest (Tian et al. 2013, 2015). Those CAFs maintaining high autocrine production of hyaluronan are more motile, whereas CAFs with fewer motile characteristics synthesized higher TGF-β1. TGF-β1 did not stimulate motility but enhance invasion and EMT markers, indicating different mechanisms to drive carcinoma progression (Costea et al. 2013). Moreover, colorectal cancer subtypes with poor prognosis share a gene program driven by TGF-β secreted by tumour stromal cells, suggesting its association to treatment resistance (Calon et al. 2015). On the other hand, it has been reported that IL-6 from CAFs enhanced TGF-β-induced EMT in non-small lung cancer cells (NSCLS). Treatment with cisplatin increased TGF-β expression, and the conditioned media from cancer cells activated fibroblasts and increased their IL-6 production, concluding that IL-6 contribute to induce a paracrine loop that intercommunicated CAFs and NSCLS, resulting in chemoresistance (Abulaiti et al. 2013; Shintani et al. 2016). Moreover, oncostatin M (OSM), an IL6 cytokine family member, induced the expression of Zeb1, Snail (SNAI1), and OSM receptor (OSMR), inducing the regulation of EMT program and conferring resistance to gemcitabine, a current first-line therapy for pancreatic ductal adenocarcinoma (Smigiel et al. 2017). Moreover, as previously mentioned hypoxia is a hallmark of solid tumours’ microenvironment and is associated to therapeutic resistance. The hypoxia-induced gene, procollagen-lysine 2-oxoglutarate 5-dioxygenase 2 (PLOD2), was induced by hypoxia conditions in biliary tract cancer cell and influence gemcitabine resistance through EMT (Okumura et al. 2018). HIF-1 has also been associated to therapy resistance. Indeed, gemcitabine resistance is associated with EMT and induction of HIF-1α in pancreatic cancer cells (Wang et al. 2014), leading to the pharmacologic manipulation of HIF-1α as novel therapeutic approach to overcome resistance. It is also important to mention that exosomes are also responsible of therapy resistance as they contain molecules that influence tumour progression. Indeed, tumour-derived exosomes may favor therapy resistance in the tumour microenvironment and induces EMT (Steinbichler et al. 2019). Indeed, exosomal miR-155 is linked to the development of drug resistance in several types of cancers via EMT, such as cisplatin resistance in oral cancer cells and in paclitaxel-resistance in gastric cancer cells (Wang et al. 2019a; Steinbichler et al. 2019; Kirave et al. 2020).

The interactions between cancer cells and immune microenvironment also play a crucial role on EMT and therapy resistance. Cancer cells produce chemokines and cytokines which recruit immune cells such as T and B lymphocytes, macrophages, neutrophils, between others (Maman and Witz 2018). For instance, in lung cancer, it has been recently reported that epigenetic suppression by Snail of the ubiquitin specific protease 4 (USP4) expression is an underlying mechanism to contribute to inflammation and therapeutic resistance by tumour-associated macrophages (Lai et al. 2020). Importantly, immunotherapy has emerged as a promising therapeutic strategy to treat cancer. Particularly, the use of immune-inhibitors targeting the interaction between PD-1 and PDL-1 or CTLA-4 have proved important benefit in cancer patients (Sharma et al. 2017; Havel et al. 2019). Although future investigations are required to deeply understand the molecular mechanism of the immune scape mechanisms, important contribution of EMT to immune escape has started to come out as well as it has been elucidated the potential use of EMT markers for immune therapy selection. The co-expression of the *N*-cadherin and Vimentin EMT markers together with PDL-1 was detected in CTCs of recurrent patients treated with nivulumab, a PD-L1 inhibitor. This evidence suggests that EMT and PDL-1 may serve to identify patients that do not respond to immunotherapy (Raimondi et al. 2017). Moreover, immunosuppression of CD8 + tumour-infiltrating lymphocytes (TIL) is linked to EMT. In this sense, microRNA-200 (miR-200) targets PD-L1. The TF Zeb1 activates EMT and transcriptionally repressed miR-200, which in consequence attenuated miR-200 repression of PD-L1 on tumour cells, leading to CD8 + T-cell immunosuppression. This important work suggests that patients on whom tumour progression is driven by EMT activators may respond to PD-L1 inhibitors (Chen et al. 2014). Moreover, EMT transcriptional scoring is a very promising strategy to determine treatment response and survival. Indeed, the EMT-transcriptional score in different tumour subtypes result in a better response to immunotherapy of those patients presenting luminal phenotype (more epithelial phenotype), than those presenting basal phenotype (mesenchymal phenotype). Importantly, different response to therapeutic administration with or without paclitaxel was observed while comparing epithelial- and mesenchymal-like phenotype in ovarian cancers, showing that mesenchymal-like tumours do not always show resistance to chemotherapy (Choi et al. 2014; Tan et al. 2014). Additionally, high score of EMT markers is related to immune expression markers, such as PDL-1 in lung adenocarcinomas or head and neck squamous cell carcinoma (Mak et al. 2016; Ock et al. 2016; Lou et al. 2016). Future investigations are required to understand the molecular mechanism by which the microenvironment may influence EMT and therapy resistance. In this context, multidisciplinary approaches including bioinformatics and pharmacology are important strategies to investigate the impact of EMT-induced therapeutic resistance.

## Bioinformatic investigation in drug resistance and in EMT

### Bioinformatic and pharmacogenomics for drug and target optimization and for drug resistance detection

In the future, bioinformatic approaches will importantly benefit the understanding of clinical relevant phenotypic programs to develop better-targeted therapies. It is becoming increasingly apparent that the use of bioinformatics and patient samples will help to study the biological impact of EMT depending on the transition dynamics, as well as to elucidate the role of EMT in drug resistance (Celià-Terrassa et al. 2018). Important publications have used mathematical or computational methods to study EMT and its potential implication in drug resistance. For instance, the use of RACIPE mathematical modeling has shown a significant negative correlation between Twist1 and E-cadherin, and a positive correlation between Twist1 and Vimentin. Moreover, Twist1 overexpression enhances genome instability in the context of EMT, thus contributing to cellular heterogeneity and potentially influencing chemoresistance (Khot et al. 2020). On the other hand, a computational approach named MAGIC (Markov affinity-based graph imputation of cells) was developed for recovering missing gene expression in single-cell data. MAGIC reveals that the majority of cells that reside in an intermediate E/M state display stem-like characteristics (van Dijk et al. 2018). Importantly, drug resistance in carcinoma cells seems to be maximal at an intermediate level of EMT-program activation (Shibue and Weinberg 2017). Foroutan et al. performed a comprehensive bioinformatics approach to show that TGFβ-driven EMT presents a low mutational burden across the TGFβ signaling pathway. Moreover, a significant variation was detected in the response of high scoring cell lines to some common cancer drugs. This scoring was applied to pan-cancer data from The Cancer Genome Atlas, showing that tumour types with high scores had significantly lower survival rates than those with low scores and carried a lower mutational burden in the TGFβ pathway. The pan-drug analysis also showed that there was no general drug resistance associated with TGFβ-induced EMT, thus reinforcing the idea of a drug-specific effect (Foroutan et al. 2017).

Pharmacogenomics is a rapidly growing field framed within genome-wide studies that aims to elucidate how human gene products (i.e. proteins) affect the response to drugs and pharmacological treatments (Roden et al. 2019). This relatively new field combines pharmacology and genomics to develop effective, safe medications and doses that can be tailored for specific tumour subtypes and specific patient risk factors (Harper and Topol 2012). It is well known that drugs can have multiple molecular targets inside our body and that the specific molecular interaction of many drugs is often unknown and can be quite variable from one individual to another. Genome and proteome-wide information associated to the drugs activity in human cells is essential to generate better maps of the molecular targets of each drug (De Las Rivas et al. 2018). Construction of this type of drug-target interaction mapping has been successful in the field of cancer genomics thanks to the possibility of testing the activity of hundreds of cancer drugs in multiple human cancer cell lines (Arroyo et al. 2020). Similar studies using genomic data combined with drugs activity are needed to elucidate at molecular level the genetic and somatic basis for inter-individual differences in drug response. The discovery of specific genetic factors that modulate the reactivity or resistance of a patient with cancer to a drug is one of the main objectives of pharmacogenomics, knowing that these factors can be critical to understand the safety, toxicity and efficacy of drugs in individual patients or in groups of patients (Lee et al. 2005; Chenoweth et al. 2020). An example of this is the discovery of multiple genetic polymorphism in gene CYP2D6, that encodes a cytochrome P450, and it is responsible for the metabolism of 25% of all drugs currently on the market. This gene presents polymorphism that significantly affect drug action. In fact, in breast cancer it has been shown that the allelic variations in CYP2D6 is a very important determinant of tamoxifen’s activity and toxicity (Huang and Ratain 2009). Another example of how pharmacogenomics can reveal resistance mechanisms is the detection, in tumours treated with EGRF inhibitors (erlotinib, gefetinib, afatinib), of the upregulation or amplification of other genes such as MET and HER2 that cause treatment failure because they replace the EGRF function (Gillis and McLeod 2016).

A key challenge that can be also addressed using pharmacogenomics is the frequent problem of ineffective response to drugs (Relling and Evans 2015; Wang and Weinshilboum 2019). The complexity drug interactions (due to the existence of multiple drug-targets, drug-drug cross reactions, target-to-target interferences, etc.) and the effect of multiple environmental factors can significantly contribute to drug inefficiency, which often also is associated to specific individual variability. In this regard, genetic factors (such as inherited variability of drug targets, drug metabolizing enzymes, and/or drug transporters) also appear to have a major impact on drug resistance (Roden et al. 2019; Chenoweth et al. 2020). In fact, specific individual resistance may be associated, for example, with the multi-drug resistance proteins (MRPs). These proteins present genetic polymorphisms that cause large differences in their expression and activity level from some individuals to others, and they are key factors in the development of resistance to different classes of anticancer drugs (Yu et al. 2007; Zhang et al. 2015).

### Cancer drug resistance: inherited or acquired

Focusing on cancer therapy, the success of target-driven anticancer drugs is usually limited by the development of several types of resistance (Rueff and Rodrigues 2016): (i) *inherited* resistance (sometimes defined as primary resistance) and (ii) *acquired* resistance (defined as secondary resistance). In both cases, resistance emerges in the context of cancer heterogeneity, either heterogeneity reflected by inter-individual differences within the same type of tumours, or heterogeneity reflected by intra-tumoural differences that reveal the phenotypic diversity of cancer cells co-inhabiting a single tumour mass (Shibue and Weinberg 2017). The first type of cancer heterogeneity (inter-individual) is often correlated with inherited primary drug resistance. The second type of cancer heterogeneity (intra-tumour) usually corresponds to secondary drug resistance, which is acquired throughout the process of tumour evolution. In both cases, pharmacogenomic studies help elucidate the molecular origin of resistance to specific anticancer drugs. For example, resistance to small-molecule tyrosine kinase inhibitors (TKIs, such as imatinib, erlotinib, gefitinib, and sorafenib), is usually acquired and shows a very different evolution in different individuals. Also genome-wide studies have shown that resistant individuals, compared to non-resistant, commonly harbor acquired somatic point mutations detected in genes NTRK1, KDR, TGFBR2 and PTPN11 and copy number alterations in CDK4, CDKN2B and ERBB2 (Gillis et al. 2017).

### EMT-induced drug resistance

EMT-induced drug resistance can be associated in most cases with the second type of cancer heterogeneity, intra-tumour, described above. Moreover, the phenotypic diversity of neoplastic cells within a tumour is considered a major driver of the development of resistance to therapy. In this context, one of the critical cell subpopulations playing a major role in the generation of drug resistance corresponds to the tumour cells that undergo EMT. Large-scale pharmacogenomics have been used to unravel how EMT can drive resistance to chemotherapeutic drugs (Hong et al. 2018). In this respect, overexpression of several genes (like integrin beta-3, ITGB3 also called CD61; and integrin beta-4, ITGB4), which promote EMT, have been directly related with chemoresistance (Li et al. 2017; Hong et al. 2018). This resistance has been linked to the activation of EMT transcription factors Snail (SNAI1) and Slug (SNAI2) in several types of cancer (Haslehurst et al. 2012). Overexpression of other EMT-inducing genes, such as Zeb1 have also been shown to closely correlate with resistance to gemcitabine, 5-fluorouracil, and cisplatin (Arumugam et al. 2009).

The specific mechanisms of how EMT induces drug resistance are still under study and may vary in different types of cancer. For example, some results observed in lung cancer (Chae et al. 2018), indicate that EMT causes a change in tumours that move them from a hot to a cold state, increasing the resistance of tumours to immunotherapy. In fact, it has been shown in non-small cell lung cancer (NSCLC) that an EMT signature is inversely associated with T-cell infiltration (Chae et al. 2018). It has also been shown that reversing EMT causes an increase in anticancer drug sensitivity (Huang and Huang 2016). Another relevant discovery in this context is that EMT often generates cells with properties of stem cells (Mani et al. 2009), which are more resistant to apoptosis and other types of programmed death, improving the capacity for self-renewal. Finally, as explained above therapeutic resistance is in many cases linked to an hybrid epithelial-to-mesenchymal phenotype (Williams et al. 2019).

## Pharmacological approaches for therapies of EMT induced drug resistance

Due to the fact that EMT is implicated in cancer metastasis and induction of drug resistance, targeting EMT may have a therapeutic value (Malek et al. 2017). As previously mentioned, interesting findings have been obtained about the correlation between either epithelial or mesenchymal status of the cell with drug resistance (Miow et al. 2014; Biddle et al. 2016). There are on-going investigations about the possibility that the specific state of these two phenomena could be reversible, among them also ours (Brozovic, unpublished data) what brought us to the thinking that possible strategy to overcome or slow down the disease progression could be targeting those specific states by targeting their regulators. There are several great review papers which are discussing in details therapeutic targets, small-molecule inhibitors of tumour plasticity as well as natural compounds which could be used for targeting tumour metastasis (Kotiyal and Bhattacharya 2014; Varghese et al. 2018; Yang et al. 2019; Feng et al. 2020). Furthermore, very nicely presented literature overview was given in a context of compounds and drugs, which target microenvironmental-induced EMT (Gao and Mittal 2012; Maman and Witz 2018). It is also known that many of EMT drivers are epigenetically regulated, by DNA methylation, histone modifications and etc., pointing out the epigenetic regulators could also be interesting therapeutic targets for overcoming EMT (Mishra and Johnsen 2014). As mentioned above, EMT is regulated by various mediators such as transcription factors, microenvironmental factors, signaling pathways, RNA‐binding proteins and miRNAs. In a line with this, there are several possible targets to overcome EMT induced drug resistance. The detailed discussion on all the targets is not possible within the limits of this review but the one investigated lately in the context of EMT induced drug resistance particularly are displayed in Table 1. Briefly, transcription factors such as Snail, Twist, Zeb or Stat3 are activated early in EMT process (Lamouille et al. 2014). Due to their importance in regulation of EMT, the inhibition of their expression or activation may be one of the ways to block EMT. Many signaling pathways, such as TGF‐β1, NF‐κB, Wnt, Akt, peroxisome proliferator activated receptor (PPAR), and Notch pathways, and the renin‐angiotensin system (RAS) contribute to the EMT. Different compounds are described as possible inhibitors of these signaling pathways for overcoming EMT (Feng et al. 2020). Recently, many miRNAs have been found to promote or suppress EMT in tumours and sensitize tumour cells to chemotherapeutics (Zhang and Ma 2012; Brozovic et al. 2015; Brozovic 2017). Moreover, post-translational EMT-regulators, such as Hakai, FBXW7 or USP27X have been emerged as new therapeutic strategies to overcome therapy resistance (Aparicio et al. 2012, 2015; Díaz and de Herreros 2016; Castosa et al. 2018; Lambies et al. 2019; Li et al. 2019; Martinez-Iglesias et al. 2020).

**Table 1:** Potential new targets to overcome EMT-induced drug resistance

EMT inducer	Proposed targets	Developed resistance	Tumour/tumour cell type	Reference
Snail (SNAI1)⇑	Snail	Cisplatin	Head and neck	Ota et al. (2016)
Twist (TWIST1) ⇑	Twist	Erlotinib Osimertinib	Non-small cell lung	Yochum et al. (2019)
Hakai (CBLL1)	Hakai	Gefitinib /cisplatin	Lung	Liu et al. (2018); Weng et al. (2019); Martinez-Iglesias et al. (2020)
FBXW7	FBXW7	Cisplatin	Colorectal	Li et al. (2019)
CAFs	ANXA2, HGF/IGF-1/ANXA2	EGFRi	Non-small cell lung	Yi et al. (2018)
CAFs	IL-6	Paclitaxel	Ovary	Wang et al. (2018)
IL-1β⇑	AKR1C1	Cisplatin	Bladder	Matsumoto et al. (2016)
IGF-1⇑	SPHK1	Paclitaxel	Lung	Wu et al. (2019b)
OSM&IL-6 (tumour microenvironment)⇑	OSM/OSMR	Gemcitabine	Pancreas	Smigiel et al. (2017)
+ TIPACF7⇑	HECTD1	Cisplatin	Breast	Duhamel et al. (2018)
TGF-β1	miR-134/-487b/-655 cluster MAGI2	Gefitinib	Lung adenocarcinoma	Kitamura et al. (2014)
TGF-β1	MCL-1	Cisplatin	Non-small cell lung	Toge et al. (2015)
TGF-β1	CXCR7	Cisplatin Etoposide	Lung	Wu et al. (2016)
TGF-β1	TGF-β1	Oxaliplatin	Colorectal	Mao et al. (2017)
TGF-β	PHD3 (- EMT regulator)	Erlotinib	Lung	Dopeso et al. (2018)
TGF-β1	ST3GAL1	Paclitaxel	Ovary	Wu et al. (2018)
TGF-β	USP27X, Snail	Cisplatin	Breast and pancreas	Lambies et al. (2019)
TGF-β1	WDR5	Paclitaxel	Breast	Punzi et al. (2019)
Hypoxia	HIF-1 (HIF1A)	Cisplatin Cetuximab Dasatinib	Head and neck squamous	Wiechec et al. (2017)
Hypoxia	PLOD2	Gemcitabine	Biliary tract	Okumura et al. (2018)
Tumour-derived exosomes	miR-155	Cisplatin	Oral cancer	Kirave et al. (2020)
iASPP⇑	miR-20, FBXL5/BTG3	Cisplatin	Cervical	Xiong et al. (2017)
CD73⇑	CD73	Trastuzumab	Lung	Turcotte et al. (2017)
FOXC2⇑	FOXC2, AKT/GSK3β	Cisplatin	Non-small cell lung	He et al. (2018)
KPNA3⇑	KPNA3, AKT/ERK	Sorafenib	Hepatocellular	Hu et al. (2019)
PRRX1⇑	SIRT1-PRRX1-KLF4-ALDH1	Paclitaxel	Breast	Shi et al. (2018)
Sema4C⇑	Sema4C, miR-31-3p	Cisplatin	Cervical	Jing et al. (2019)
SNHG3⇑	miR-128/CD151	Sorafenib	Hepatocellular	Zhang et al. (2019)
TYRO3⇑	Snail	Paclitaxel Oxalilatin 5-fluorouracil	Colon	Chien et al. (2016)
miR-93⇑	PTEN	Doxorubicin	Breast	Chu et al. (2017)
miR-296-3p⇑	PRKCA-FAK-RAS-cMYC	Cisplatin	Lung adenocarcinoma	Fu et al. (2017)
miR-216a/-217⇑	PTEN, SMAD	Sorafenib	Liver	Xia et al. (2013)
miR-574-3p⇓	Zeb1	Cisplatin	Gastric	Wang et al. (2019b)
miR-509 and miR-1243⇓	CDH1	Gemcitabine	Pancreas	Hiramoto et al. (2017)

*AKR1C1*, aldo–keto reductase family 1 member C1; *ANXA2*, annexin A2; *CBF1*, centromere-binding protein 1; *CDH1*, E-cadherin; *COX-2*, cyclooxygenase-2; *CXCR7*, C-X-C chemokine receptor type 7; *FAK*, focal adhesion kinase; *FBXW7*, F-Box E3 ubiquitin-ligase; *Hakai*, HYB domain E3 ubiquitin, ligase; *HectD1*, E3 ubiquitin-ligase; *HGF*, hepatocyte growth factor. *IGF-1*, insulin like growth factor 1; *MCL-1*, myeloid leukemia cell differentiation protein; *Oct4*, octamer-binding transcription factor 4; *OSM/OSMR*, oncostatin-M/oncostatin M receptor; *PARP3*, poly(ADP-Ribose) polymerase family member 3; *PHD3*, prolyl-4-hydroxylase domain 3; *PLOD2*, procollagen-lysine,2-oxoglutarate 5-dioxygenase 2; *PRKCA*, protein kinase C alpha; *PTEN*, phosphatase and tensin homolog; *Ras*, rous sarcoma virus; *SHKBP1*, SH3KBP1 binding protein 1; *SMAD*, mothers against decapentaplegic homolog 1; *SOX2*, sex determining region Y-box 2 (or SRY); *SphK1*, sphingosine kinase 1; *ST3GAL1*, ST3 beta-galactoside alpha-2; *USP27X*, X-linked ubiquitin carboxyl-terminal hydrolase 27, deubiquitinase; *WDR5*, WD repeat domain

Some novel mediators specifically involved in EMT-induced drug resistance are also shown in Table 1. Inhibitor of apoptosis-stimulating protein of p53 (iASPP), which was previously confirmed as EMT inducer, promotes miR-20a expression in a p53-dependent manner. MiR-20 upregulation induces EMT and cisplatin resistance via F-box and leucine rich repeat protein 5/BTG anti-proliferation factor 3 (FBXL5/BTG3) signaling in cervical cancer HeLa and SiHa cell lines (Dong et al. 2016; Xiong et al. 2017). Forkhead box protein C2 (FOXC2) was shown to promote resistance to the same drug by induction of EMT in non-small cell lung cancer A549 cells by activating v-akt murine thymoma viral oncogene homolog 1/ Glycogen synthase kinase 3 (AKT/GSK3) signaling pathway and increased expression of Snail (He et al. 2018). Cisplatin resistance was also induced by Sema4C, and upregulation of miR-31-3p which reversed EMT-mediated biological functions in human cervical cancer HeLa, Caski, Siha and C33a cell lines (Jing et al. 2019). It was proposed that depleting sirtuin 1 (SIRT1) accelerates the degradation of paired related homeobox 1 (PRRX1) and disinhibits kruppel-like factor 4 (KLF4) transcription, leading to a partial MET, occurrence of aldehyde dehydrogenase 1 (ALDH1)-positive cancer stem cells, distant metastases and resistance to paclitaxel. Reduced nuclear level of SIRT1-PRRX1 axis is positively correlated with lung metastasis of breast cancer (Shi et al. 2018). Tyrosine-protein kinase receptor TYRO3 is overexpressed in the early stage of colon cancer development and aberrant expression of TYRO3 promotes tumourigenesis and induces EMT through the regulation of SNAI1. Blocking TYRO3 signaling by human anti-TYRO3 antibody ameliorates cancer malignancy and increased sensitivity to paclitaxel, oxaliplatin and 5-fluorouracil in different colon cancer cell lines (Chien et al. 2016). Expression of ectonucleotidase CD73 by tumour, stromal and immune cells is associated with immune suppression (Allard et al. 2017). But it was shown that expression of CD73 is associated with extracellular matrix organization, TGF-β genes, EMT, hypoxia-inducible factor-1 (HIF-1) as well and is mediating resistance to trastuzumab in human breast cancer (Turcotte et al. 2017). A novel KPNA3-AKT-ERK-TWIST signaling cascade that promotes EMT and mediates sorafenib resistance was described in human hepatocellular cell lines Huh7 and HepG2 cells (Hu et al. 2019). Moreover, sorafenib resistance was induced by SNHG3 overexpression in several human hepatocellular cells PLC/PRF/5, Hep3B, HepG2, MHCC97L, Huh7, SMMC‐7721, and HCCLM3 EMT via miR-128/CD151 cascade activation (Zhang et al. 2019).

## Conclusions

The heterogeneity and plasticity of EMT phenotype are features not only involved in metastasis, but also in drug resistance. In recent years, new molecular insights have come out with the implication of EMT in drug resistance thanks to the in vitro and in vivo studies in preclinical tumour models and clinical settings. The knowledge of intermediate E/M states, that represent more properly the reality within the tumour, together with the influence of tumour microenvironment and cellular stemness, has opened new understanding regarding to the impact of the EMT to therapy resistance. Moreover, new therapeutic strategies based on epithelial plasticity have been proposed to overcome therapy resistance. The development of plasticity inhibitors may have a great potential in cancer treatment as this type of drugs may prevent both drug resistance and cancer metastasis. Compounds targeting regulators of this plasticity could also work well with chemotherapy or targeted therapy drugs improving in that way the clinical outcomes of cancer patients. The integration of bioinformatics, pharmacogenomics and chemical genomic data will be crucial to identify both therapeutic targets and novel chemosensitizing drugs to overcome resistance to multiple chemotherapies. A wide range of targets associated with EMT is expected to be elucidated in the future, thus allowing to overcome therapy resistance. This will allow paving an alternative path for drug discovery even for proteins that cannot be pharmacological targeted nowadays.

## Data Availability

All the data obtained and/or analyzed during the current study were available from the corresponding authors on reasonable request.

## Abbreviations

ECM: Extracellular matrix
CAFs: Cancer-associated fibroblast
EMT: Epithelial-to-mesenchymal transition
E/M: Epithelial/mesenchymal states
HIF: Hipoxia-iducible factors
MDR: Multidrug resistance
NSCLC: Non-small cell lung cancer
SCLC: Small cell lung cancer
TAMs: Tumour associated macrofges
TFs: Transcription factors
TME: Tumour microenvironment

## Declarations

### Author details

A.F. is group leader of the Epithelial Plasticity and Metastasis Group at the Instituto de Investigación Biomédica de A Coruña (INIBIC), Complexo Hospitalario Universitario de A Coruña (CHUAC), Sergas, Universidade da Coruña (UDC), Spain. Expert in epithelial-mesenchymal transition in cancer metastasis and drug resistance. J.D.L.R is head of the Bioinformatics and Functional Genomics Group at the Cancer Research Center (CiC-IBMCC, CSIC/USAL/IBSAL), Consejo Superior de Investigaciones Cientificas (CSIC) and University of Salamanca (USAL), Salamanca. Vice Chair of STRATAGEM “New diagnostic and therapeutic tools against multidrug resistant tumours”, a European COST action (European Cooperation in Science and Technology). Expert in Bioinformatics and Functional Genomics and cancer drug resistance. I.P.W is head of the Laboratory of Tumour Microenvironment & Metastasis Research at the The Shmunis School of Biomedicine and Cancer Research, George S. Wise Faculty of Life Sciences, Tel-Aviv University, Tel Aviv, Israel. Expert in the interactions between tumour cells and tumour microenvironment. A.B. is senior research associate and group leader in Division of Molecular Biology, Ruđer Bošković Institute, Zagreb, Croatia. Expert in molecular mechanisms of drug resistance and drug-induced epithelial-mesenchymal transition.
